# Causal associations of circulating adiponectin with cardiometabolic diseases and osteoporotic fracture

**DOI:** 10.1038/s41598-022-10586-1

**Published:** 2022-04-23

**Authors:** Muzi Zhang, Xiaojun Chen, Yong Zhu, Lifeng Yin, Zhengxue Quan, Yunsheng Ou, Bin He

**Affiliations:** 1 Department of Orthopedics, The First Affiliated Hospital of Chongqing Medical University, No. 1 Yi Xue Yuan Road, Yuzhong District, Chongqing, 400016 China; 2 Department of Orthopedics, The Affiliated Traditional Chinese Medicine Hospital of Southwest Medical University, Luzhou, Sichuan China

**Keywords:** Diseases, Endocrinology, Health care

## Abstract

Circulating adiponectin shows some relationships with the occurrence of cardiometabolic diseases and osteoporotic fracture, but little is known about their causal associations. This two-sample Mendelian randomization (MR) study aims to explore the causal roles of circulating adiponectin in cardiometabolic diseases and osteoporotic fracture. We used 15 single nucleotide polymorphisms associated with circulating adiponectin as the instrumental variables. Inverse variance weighted, weighted median and MR-Egger regression methods were applied to study the causal associations. The results found that high circulating adiponectin was causally associated with reduced risk of type 2 diabetes (beta-estimate: −0.030, 95% CI: −0.048 to −0.011, SE: 0.009, *P*-value = 0.002) and may be the risk factor of coronary artery disease (beta-estimate: 0.012, 95% CI: 0.001 to 0.023, SE: 0.006, *P*-value = 0.030). No causal associations were seen between circulating adiponectin and other outcomes including heart failure, atrial fibrillation, cerebral ischemia, intracerebral hemorrhage or osteoporotic fracture. This study found the potential causal roles of high circulating adiponectin in reduced risk of type 2 diabetes and increased risk of coronary artery disease, which may help prevent and treat these two diseases.

## Introduction

Excess adiposity is widely accepted as the risk factor to cause many diseases such as cardiometabolic diseases and osteoporosis^1–4^. In patients with obesity, continued infiltration of immune cells (e.g. macrophages) into adipose tissue affects the secretion of many adipokines such as adiponectin and leptin^5^. Especially, adiponectin has emerged as an increasingly important factor because of its potential in protecting against type 2 diabetes, anti-inflammatory and anti-atherogenic effects^6–8^.

Observational studies reported that circulating adiponectin was associated with cardiometabolic diseases and osteoporotic fracture, but may suffer from confounding factors and reverse causality^9–14^. Their causal associations are not clear, and Mendelian randomization (MR) study has become one effective and powerful approach to explore the causal relationships between exposure phenotype and outcome phenotype^15–17^. Furthermore, two-sample MR analysis is able to greatly improve statistical power of MR study^17–20^.

Circulating adiponectin, cardiometabolic diseases and osteoporosis are highly polygenic traits according to the genome-wide association studies (GWASs)^19,21–24^. Adiponectin has important potential in regulating inflammatory responses which are associated with the modulation of cardiovascular diseases (e.g. coronary artery disease, heart failure and atrial fibrillation), type 2 diabetes, cerebral ischemia and osteoporosis^25–31^. Cardiometabolic diseases and osteoporosis have robust connection of pathogenesis. In addition, our recent MR study provides robust evidence that high circulating adiponectin is causally associated with the increased incidence of osteoporosis and low bone mineral density (BMD)^32^, but it is unknown whether circulating adiponectin affects the occurrence of osteoporotic fracture. Therefore, this two-sample MR study aims to study the causal influence of circulating adiponectin on the incidence of cardiometabolic diseases and osteoporotic fracture.

## Methods

### Data source of circulating adiponectin

One recent GWAS meta-analysis aimed to find the adiponectin-associated SNP variants, and included 25 independent studies and 67,739 adult individuals of the following ancestries: (1) European (n ≤ 60,465), (2) East Asian (n ≤ 2568), (3) African American (n ≤ 3271) and (4) Hispanic (n ≤ 1435)^33^. Different methods were used to measure adiponectin levels and comprised enzyme-linked immunosorbent assay (ELISA), radioimmunoassay (RIA), and dissociation-enhanced lanthanide fluoroimmunoassays (DELFIA). The unit of adiponectin levels was mg/mL. The results was adjusted for age, sex, body mass index (BMI) and principal components (PCs) that may cause population stratification^33^.

Initially, 18 SNPs were identified to have robust association with circulating adiponectin (*P* < 5 × 10^−8^, Supplementary Table S1). Linkage disequilibrium (LD) between selected SNPs was calculated using European samples from the 1000 Genomes project. Three SNPs (rs3087866, rs145119400 and rs3865188) were excluded due to high LD (r^2^ ≥ 0.001). Finally, we selected 15 SNPs as instrumental variables of circulating adiponectin (Supplementary Table S2).

### Outcome data sources

Several largest GWASs reported the summary-level data associated with genetic associations with outcomes (Table 1). Briefly, we studied cardiometabolic diseases including type 2 diabetes (898,130 individuals) from DIAGRAM^34^, coronary artery disease (547,261 individuals) from UK Biobank and CARDIoGRAMplusC4D^35^, heart failure (977,323 individuals) from UK Biobank^36^, atrial fibrillation (587,446 individuals) from one large meta-analysis^37^, cerebral ischemia (401,937 individuals) and intracerebral hemorrhage (399,717 individuals) from UK Biobank^38^. Osteoporotic fracture (426,795 individuals) were defined as any fracture apart from the fracture of skull, face, hands, feet, and pathological fractures due to malignancy, atypical femoral fractures, periprosthetic and healed fracture^39^. All participants in these GWASs were all from European descent except those with atrial fibrillation from predominantly European descent (mixed descents). Supplementary Table S2 demonstrated the SNP summary statistics related to circulating adiponectin and each outcome.

**Table 1:** Details of studies and datasets used for analyses.

	Traits	Samples size	Population	Consortium or cohort study (Link URL)
Exposure	Adiponectin	67,739	Predominant European (Mixed)	Meta-analysis of 25 studies
Cardiometabolic diseases	Type 2 diabetes	8,98,130	European	DIAGRAM (http://diagram-consortium.org)
	Coronary artery disease	5,47,261	European	UK Biobank and CARDIoGRAMplusC4D (https://cvd.hugeamp.org/)
	Heart failure	9,77,323	European	UK Biobank (http://www.broadcvdi.org/)
	Atrial fibrillation	5,87,446	Predominant European (Mixed)	Meta analysis of more than 50 studies (http://www.broadcvdi.org/)
	Cerebral ischemia	4,01,937	European	UK Biobank (https://www.leelabsg.org/resources)
	Intracerebral hemorrhage	3,99,717	European	
Osteoporosis	Osteoporotic fracture	4,26,795	European	GEFOS (http://www.gefos.org)

### Statistical analyses

We used inverse variance weighted (IVW) meta-analysis of Wald ratio, weighted median and MR-Egger regression methods to assess the causal influence of circulating adiponectin on each outcome. The intercept term in MR-Egger regression was useful to assess the directional horizontal pleiotropy. Cochran’s Q analysis was applied to assess the heterogeneity^19^. Q statistic represented a chi-square distribution with m−1 degrees of freedom under the null hypothesis of homogeneity and its equation was presented as:

$$ Q = \sum\limits_{k = 1}^{m} {w_{k} \left( {\hat{\beta }_{XY}^{(k)} - \mu_{F} } \right)^{2} } $$

where m was the number of estimates to be pooled, $w_{k}$ was the weight for the estimate $\hat{\beta }_{XY}^{(k)}$ and represented the precision (reciprocal of the variance) of the estimate and μ_F_ was a weighted mean estimate calculated as $\mu_{{\text{F}}} = \sum {w_{k} \hat{\beta }_{XY}^{(k)} /\sum {w_{k} } }$.

I^2^ index was defined as the percentage of total variation in the estimates explained by heterogeneity, and was calculated as:

$$ I^{2} = \left\{ {\begin{array}{*{20}l} {\frac{Q - (m - 1)}{Q} \times 100,} \hfill & {{\text{for}}} \hfill & {Q \ge m - 1} \hfill \\ {0,} \hfill & {{\text{for}}} \hfill & {Q < m - 1} \hfill \\ \end{array} } \right. $$

Heterogeneity *p*-value < 0.05 indicated significant heterogeneity, while pleiotropy *p*-value < 0.05 suggested the presence of pleiotropic SNPs^40^. MR-PRESSO analysis attempted to find the pleiotropic SNPs and then reduce heterogeneity in the causal estimation by removing SNP outliers^41^.

All methods were carried out in accordance with relevant guidelines and regulations. Because this MR study was conducted by using publicly available GWAS summary data, ethical approval and informed consent obtained from all subjects could be found in the original publications. All analyses were performed in R V.4.0.4 by using the R packages of ‘MendelianRandomization’^42^, ‘TwoSampleMR’^43^ and ‘MR-PRESSO’ ^44^.

## Results

### Cardiometabolic diseases

**Figure 1:**
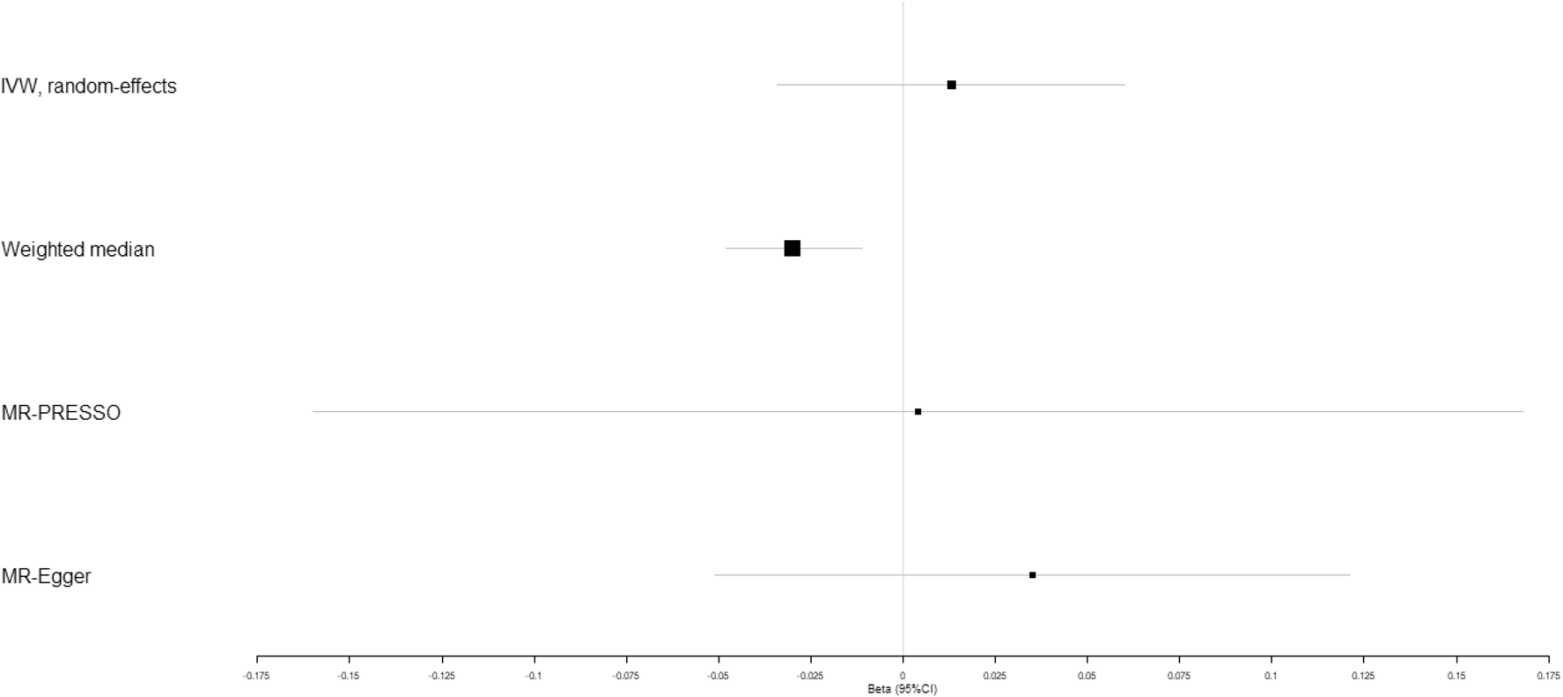
Beta (95% CIs) for causal influence of circulating adiponectin on type 2 diabetes through multiple analyses.

We evaluated the causal effect of circulating adiponectin on type 2 diabetes, coronary artery disease, heart failure, atrial fibrillation, cerebral ischemia and intracerebral hemorrhage using multiple MR methods (Table 2). According to the weighted-median analysis, genetically high circulating adiponectin played a significant causal role in reduced risk of type 2 diabetes (beta-estimate: −0.030, 95% CI: −0.048 to −0.011, SE:0.009, *P*-value = 0.002), but it was not supported by IVW analysis (*P*-value = 0.590) or MR-Egger result (*P*-value = 0.426, Fig. 1 and Table 2). Scatter plot of the association between circulating adiponectin and Type 2 diabetes was shown in Supplementary Fig. S1.

**Table 2:** Mendelian randomization estimates of adiponectin on outcomes.

Variables	IVW							Weighted median			
	Estimate	SE	95% CI	*P*-value	Q value	I^2^ (%)	Heterogeneity *P* value	Estimate	SE	95% CI	*P*-value
Type 2 diabetes	0.013	0.024	−0.034, 0.060	0.590	410.127	96.60	0.000	−0.030	0.009	−0.048, 0.011	0.002
Coronary artery disease	0.006	0.007	−0.009, 0.020	0.439	74.219	81.10	0.000	0.010	0.006	−0.001, 0.021	0.085
Heart failure	0.001	0.006	−0.012, 0.014	0.868	25.398	44.90	0.031	0.002	0.007	−0.011, 0.015	0.787
Atrial fibrillation	0.001	0.007	−0.012, 0.014	0.929	33.463	58.20	0.003	−0.007	0.006	−0.020, 0.005	0.240
Cerebral ischemia	−0.002	0.016	−0.033, 0.030	0.924	14.543	3.70	0.410	0.006	0.021	−0.034, 0.046	0.766
Intracerebral hemorrhage	−0.035	0.038	−0.109, 0.039	0.360	19.516	28.30	0.146	−0.026	0.045	−0.114, 0.062	0.567
Osteoporotic fracture	0.007	0.005	−0.003, 0.016	0.173	21.500	34.90	0.090	0.006	0.006	−0.006, 0.018	0.297

Variables	MR-Egger										
	Estimate	SE	95% CI	*P*-value	Intercept	SE	95% CI	Pleiotropy *P* value			
Type 2 diabetes	0.035	0.044	−0.051, 0.121	0.426	−0.013	0.021	−0.054, 0.028	0.545			
Coronary artery disease	0.004	0.013	−0.022, 0.030	0.771	0.001	0.006	−0.012, 0.013	0.879			
Heart failure	−0.003	0.012	−0.026, 0.020	0.787	0.002	0.006	−0.009, 0.013	0.663			
Atrial fibrillation	−0.019	0.010	−0.040, 0.001	0.062	0.011	0.005	−0.002, 0.021	0.020			
Cerebral ischemia	−0.032	0.028	−0.087, 0.024	0.261	0.017	0.013	−0.009, 0.044	0.197			
Intracerebral hemorrhage	−0.030	0.070	−0.168, 0.107	0.664	−0.002	0.033	−0.068, 0.063	0.943			
Osteoporotic fracture	0.000	0.009	−0.017, 0.017	0.970	0.004	0.004	−0.004, 0.012	0.379			

Q statistic represents a chi-square distribution with m-1 degrees of freedom under the null hypothesis of homogeneity. I^2^ index is defined as the percentage of total variation in the estimates explained by heterogeneity. Heterogeneity *p*-value < 0.05 indicates significant heterogeneity, while pleiotropy *p*-value < 0.05 suggests the presence of pleiotropic SNPs.

**Figure 2:**
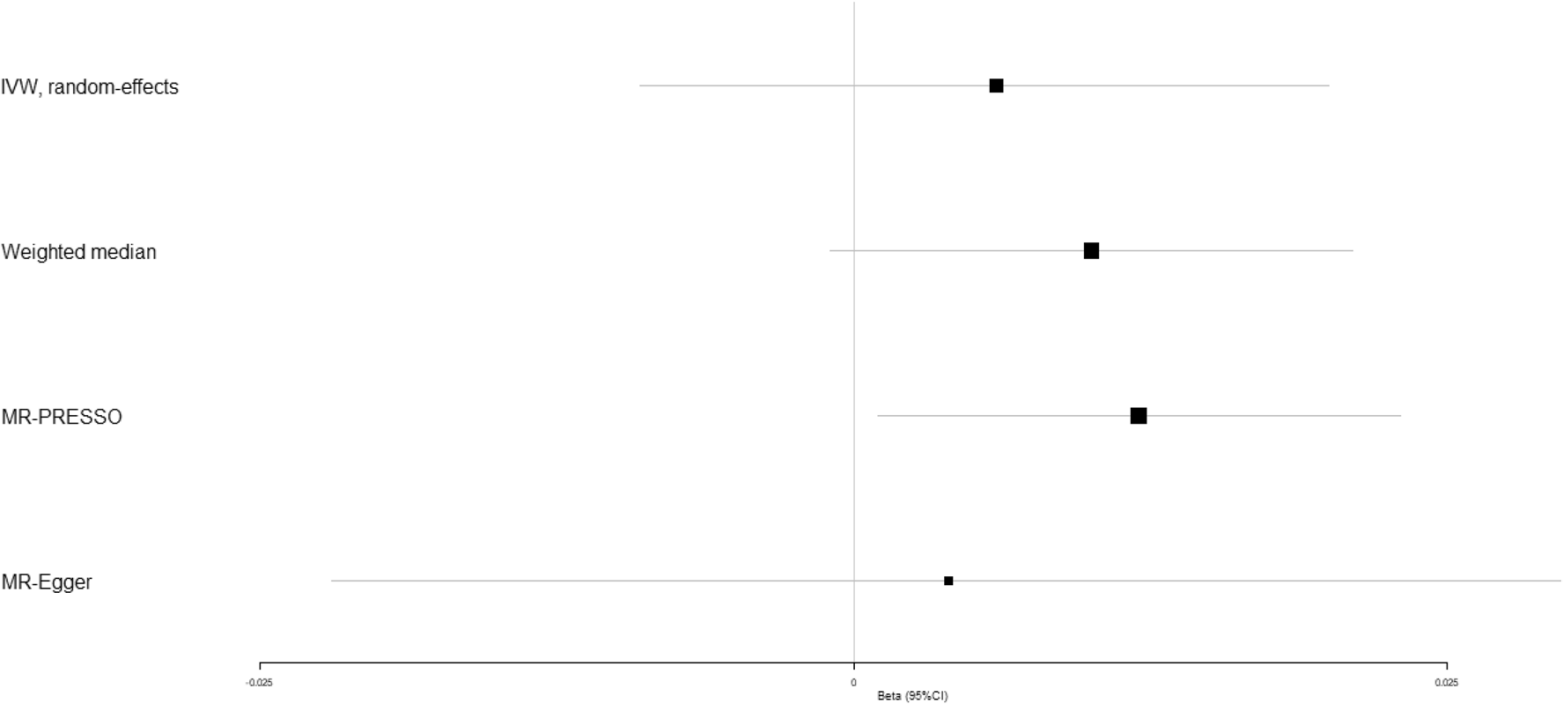
Beta (95% CIs) for causal influence of circulating adiponectin on coronary artery disease through multiple analyses.

**Figure 3:**
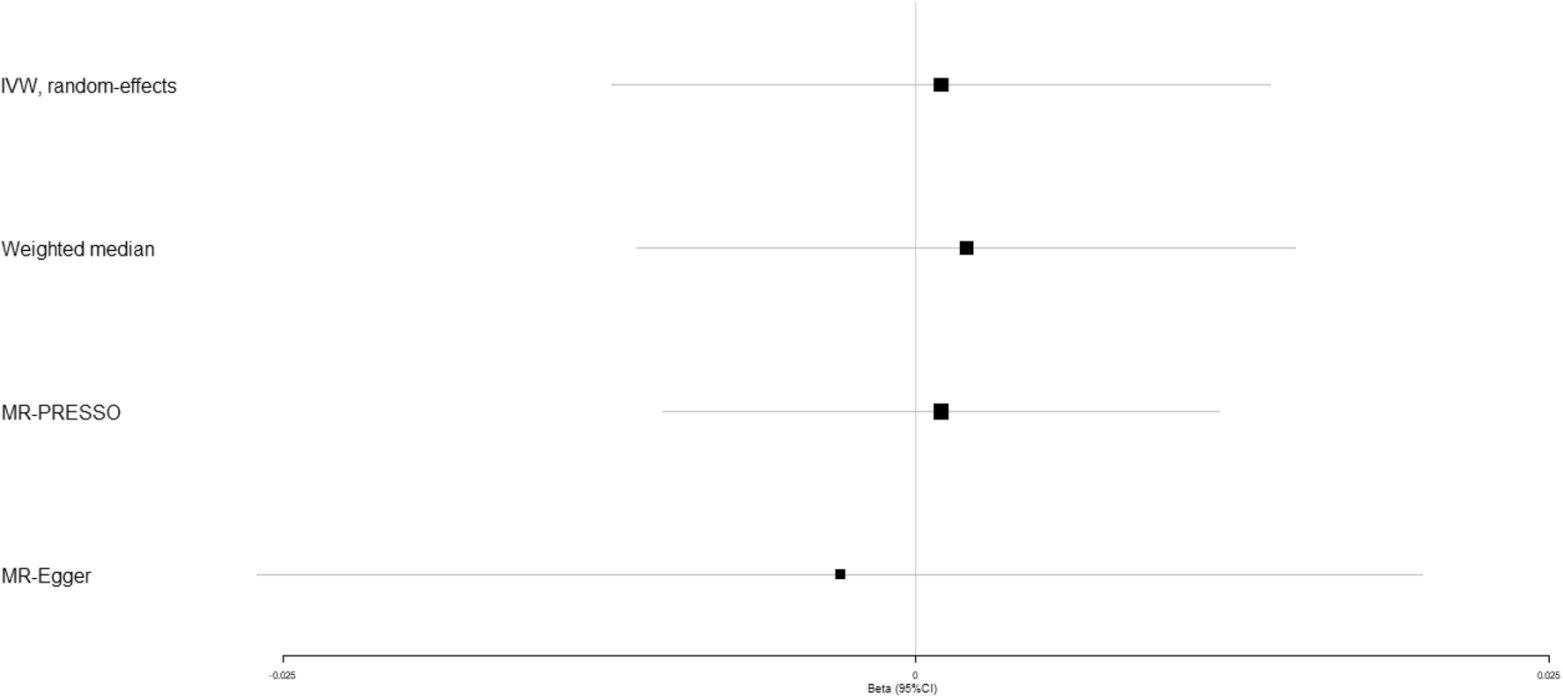
Beta (95% CIs) for causal influence of circulating adiponectin on heart failure through multiple analyses.

**Figure 4:**
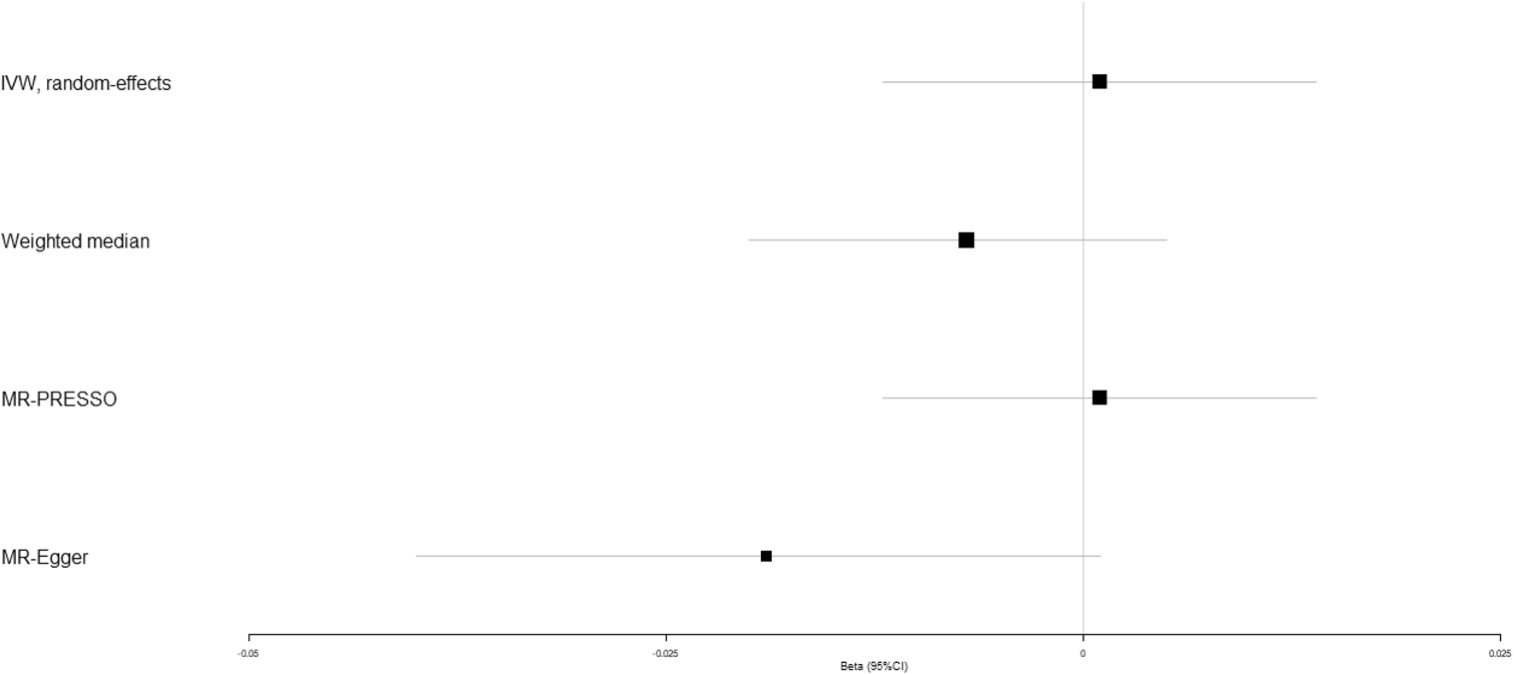
Beta (95% CIs) for causal influence of circulating adiponectin on atrial fibrillation through multiple analyses.

**Figure 5:**
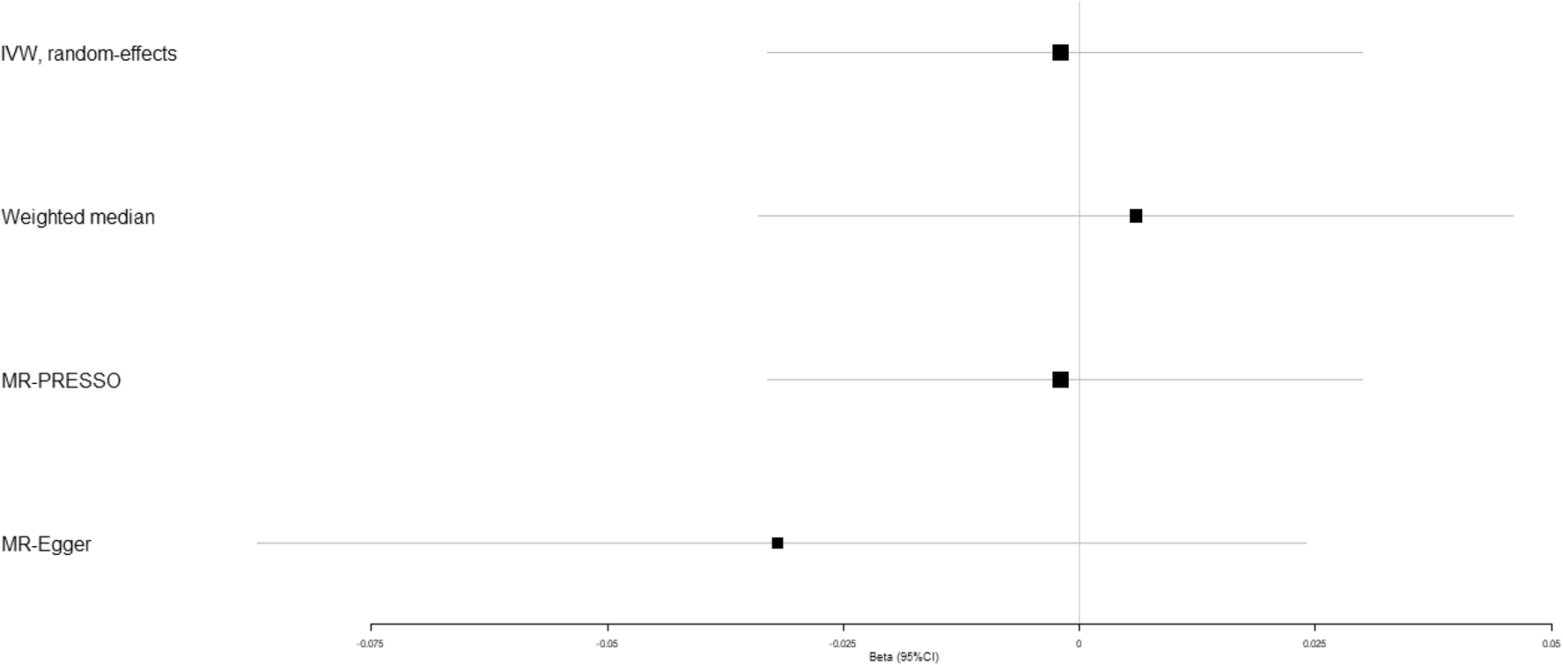
Beta (95% CIs) for causal influence of circulating adiponectin on cerebral ischemia through multiple analyses.

**Figure 6:**
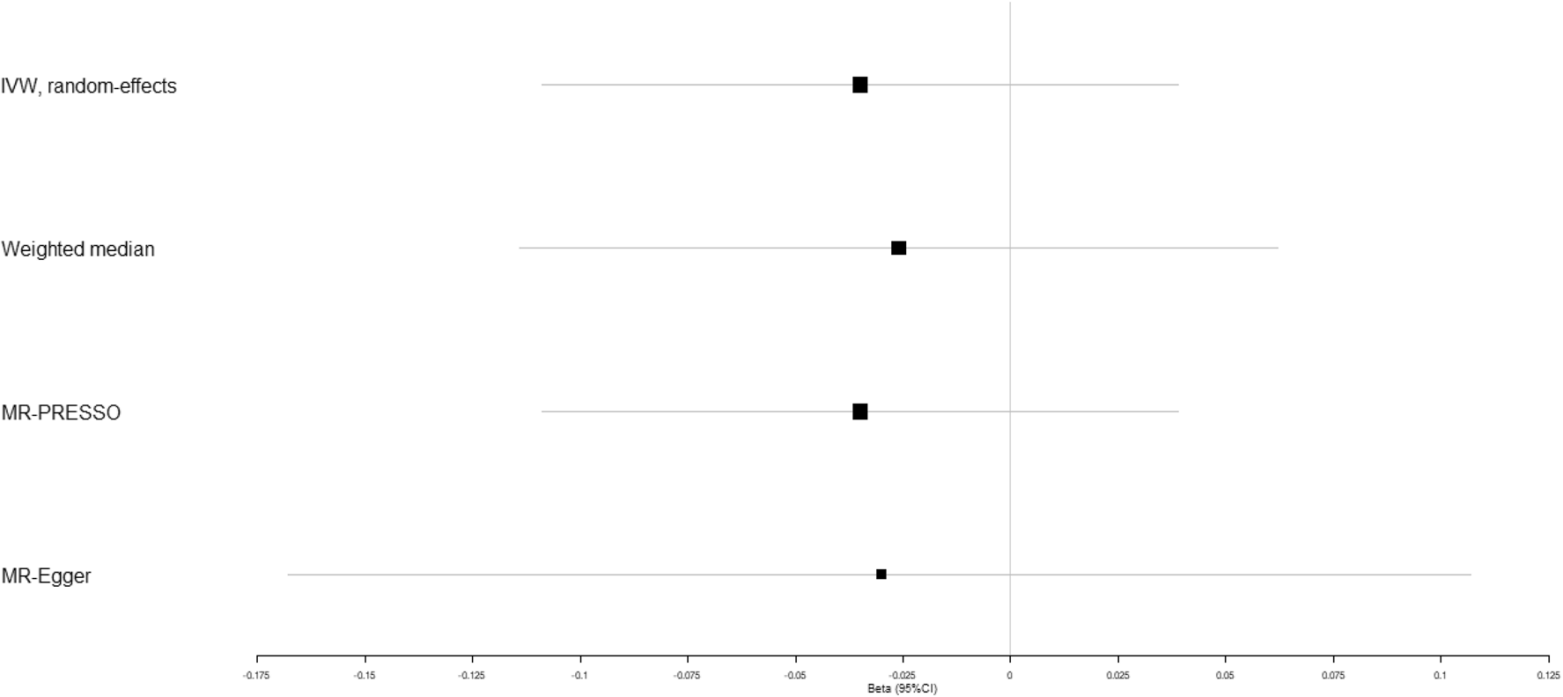
Beta (95% CIs) for causal influence of circulating adiponectin on intracerebral hemorrhage through multiple analyses.

In addition, IVW analyses showed that circulating adiponectin demonstrated no obviously causal impact on coronary artery disease (beta-estimate: 0.006, 95% CI: −0.009 to 0.020, SE:0.007, *P*-value = 0.439, Fig. 2 and Supplementary Fig. S2), heart failure (beta-estimate: 0.001, 95% CI: −0.012 to 0.014, SE:0.006, *P*-value = 0.023, Fig. 3 and Supplementary Fig. S3), atrial fibrillation (beta-estimate: 0.001, 95% CI: −0.012 to 0.014, SE: 0.007, *P*-value = 0.929, Fig. 4 and Supplementary Fig. S4), cerebral ischemia (beta-estimate: −0.002, 95% CI: −0.033 to 0.030, SE:0.016, *P*-value = 0.924, Fig. 5 and Supplementary Fig. S5) or intracerebral hemorrhage (beta-estimate: −0.035, 95% CI: −0.109 to 0.039, SE: 0.038, *P*-value = 0.173, Fig. 6 and Supplementary Fig. S6), and these results were confirmed by the weighted-median and MR-Egger analyses (*P* > 0.05, Table 2, Fig. 2–6 and Supplementary Figs. S2–S6).

### Osteoporotic fracture

**Figure 7:**
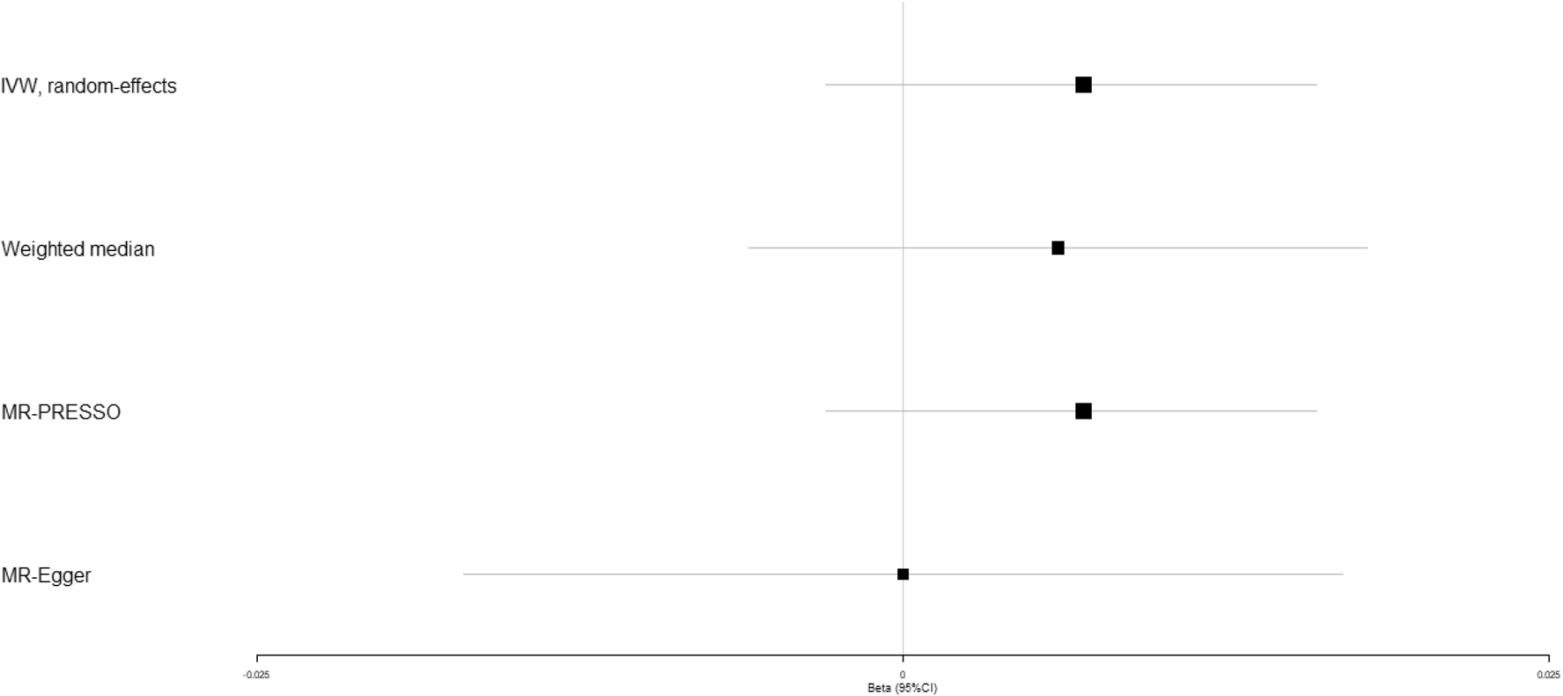
Beta (95% CIs) for causal influence of circulating adiponectin on osteoporotic fracture through multiple analyses.

Previous studies have demonstrated that high circulating adiponectin is a risk factor of osteoporosis^12,13^, but it remains elusive whether circulating adiponectin affects the occurrence of osteoporotic fracture. IVW analysis unraveled that circulating adiponectin showed no causal role in the risk of osteoporotic fracture (beta-estimate: 0.007, 95% CI: −0.003 to 0.016, SE: 0.005, *P*-value = 0.173), which was also confirmed in weighted-median analysis (*P*-value = 0.297) and MR-Egger analysis (*P*-value = 0.970, Table 2 and Fig. 7). Scatter plot of the association between circulating adiponectin and osteoporotic fracture was shown in Supplementary Fig. S7.

### Evaluation of assumptions and sensitivity analyses

Little evidence of directional pleiotropy was revealed for all models except the association between circulating adiponectin and atrial fibrillation (MR-Egger intercept *P*-value = 0.020, Table 2). There was significant heterogeneity for type 2 diabetes, coronary artery disease and atrial fibrillation. Thus, MR-PRESSO method was performed to find the SNP outliers, including 13 outliers (rs2791552, rs2943641, rs2276853, rs13303, rs13133548, rs4311394, rs3735080, rs10861661, rs7134375, rs11057405, rs11057353, rs2925979, rs4805885) for type 2 diabetes, 3 outliers (rs2943641, rs2925979, rs4805885) for coronary artery disease and one outlier (rs10861661) for heart failure (Table 3).

**Table 3:** Mendelian randomization estimates between adiponectin and outcomes after excluding outliers detected by MR-PRESSO.

Outcomes	Estimate	SE	95% CI	*P*-value
Type 2 diabetes excluding 13 outliers (rs2791552, rs2943641, rs2276853, rs13303, rs13133548, rs4311394, rs3735080, rs10861661, rs7134375, rs11057405, rs11057353, rs2925979, rs4805885)	0.004	0.084	0.160, 0.168	0.961
Coronary artery disease excluding 3 outliers (rs2943641, rs2925979, rs4805885)	0.012	0.006	0.001, 0.023	0.030
Heart failure excluding 1 outlier (rs10861661)	0.001	0.005	0.010, 0.012	0.885

After excluding these SNP outliers, high circulating adiponectin showed the causal effect on increased risk of coronary artery disease (beta-estimate: 0.012, 95% CI: 0.001 to 0.023, SE:0.006, *P*-value = 0.030, Fig. 2 and Table 3). In addition, the MR association between circulating adiponectin with other outcomes were not changed after excluding the outlying SNP variants (Table 3).

## Discussion

In this two-sample MR study, we found that high circulating adiponectin was causally associated with reduced risk of type 2 diabetes, but may be the risk factor of coronary artery disease. In addition, no causal roles of circulating adiponectin were revealed in the incidence of heart failure, atrial fibrillation, cerebral ischemia, intracerebral hemorrhage or osteoporotic fracture. These findings suggested that circulating adiponectin levels may provide new insights to prevent and treat type 2 diabetes and coronary artery disease.

Many studies report the associations between circulating adiponectin and insulin resistance, lipid levels, inflammatory markers, atherosclerosis biomarkers, type 2 diabetes and cardiovascular diseases, but their causal relationships remain elusive^25,45–47^. Observational studies reported the significantly inverse relationship between circulating adiponectin and fasting-insulin level^48,49^, and the close correlation between adiponectin levels and the incidence of type 2 diabetes was found in one population-based study^50^. In contrast, another study documented no association between circulating adiponectin and risk of type 2 diabetes^51^.

Considering these inconsistent results, two-sample MR study has become an increasingly important approach to explore risk factors of diseases^19^. One recent MR study included GWAS meta-analysis of circulating adiponectin levels (n = 39,883) and GWAS meta-analysis of type 2 diabetes (n = 659,316). The results found no causal effect of circulating adiponectin levels on the risk of type 2 diabetes^52^. More large-scale patient population were involved in our two-sample MR study, including the GWAS meta-analyses of circulating adiponectin levels (n = 67,739) and type 2 diabetes (n = 89,8130). Our research results revealed the high circulating adiponectin levels displayed a causal role in the decreased risk of type 2 diabetes. This protective effect of adiponectin on type 2 diabetes was attributed to anti-inflammatory properties and improvement in insulin sensitivity^53^, which were mediated by suppression of tumour necrosis factor alpha (TNF-α)^54^, inhibition of nuclear factor kappa B (NF-κB) in macrophages^55^, improved expression of interleukin-10 (IL-10) and promotion to macrophage transformation from M1 to M2^56^.

Various studies reported that high circulating adiponectin levels were associated with low risk of cardiovascular diseases^57,58^, which were in contrast to other studies^59–61^. In addition, high circulating adiponectin levels may be the risk factor to increase mortality in patients with coronary artery disease^62^. One recent MR study included the GWASs associated with adiponectin (n = 39,883)^63^ and coronary artery disease (n = 184,305)^64^. The results found that high adiponectin was unlikely to be the risk factor of coronary artery disease^65^. However, our MR study included much larger-scale populations (i.e. 67,739 individuals related to adiponectin^33^ and 547,261 individuals associated with coronary artery disease^35^). After excluding these SNP outliers detected by MR-PRESSO method, high circulating adiponectin showed the causal effect on increased risk of coronary artery disease (beta-estimate: 0.012, 95% CI: 0.001 to 0.023, SE:0.006, *P*-value = 0.030, Fig. 2 and Table 3). These suggested that high circulating adiponectin may be one risk factor of coronary artery disease.

Adiponectin is almost exclusively produced by adipocytes, and its secretion is strongly dependent on cyclic guanosine monophosphate (cGMP)-dependent protein kinase which is activated in response to natriuretic peptide binding to specific receptor^66^. Thus, high adiponectin may be associated with high natriuretic peptide, which is a risk factor of coronary artery disease^67^. In addition, overproduction of adiponectin improves cardiac hypertrophy and cardiac function, and protect against ischemic/reperfusion injury in experimental models^68,69^.

Osteoporosis widely occurs in aging people and post-menopausal women, and is widely accepted to increase the incidence of osteoporotic fracture^70,71^. High adiponectin levels was documented to be a risk factor of osteoporotic fracture, but the positive finding may be affected by potential confounding factors and reverse causality^14^. Our recent MR study found that high circulating adiponectin has significantly causal impact on low BMD^32^, but the causal association between circulating adiponectin and osteoporotic fracture remains elusive. Our multiple analyses confirmed no causal relationship between circulating adiponectin and osteoporotic fracture. In addition, there is limited evidence of associations between circulating adiponectin and heart failure, atrial fibrillation, cerebral ischemia and intracerebral hemorrhage.

We should consider several strengths. Our MR study includes large-scale populations in order to investigate the causal effect of circulating adiponectin on cardiometabolic diseases and osteoporotic fracture. We use strong SNPs as instrumental variables (*P* < 5 × 10^−8^), and excluded SNPs in high LD. Multiple sensitivity analyses are used to test the influence of pleiotropy on causal estimates. To increase the reliability of our results, the outlier variants identified by the MR-PRESSO test are removed and causal estimates are recalculated. There are also several important limitations. Firstly, serum adiponectin is measured by various methods including ELISA, RIA and DELFIA, which may produce some heterogeneity. Secondly, participants in the summary GWASs are of predominantly European descent, but we can not perform the MR analyses based on different ancestries. Thus, our findings may not be fully representative of the whole population. Thirdly, the MR association between high serum adiponectin and decreased risk of type 2 diabetes is significant according to the weighted-median analysis, which is not supported by IVW, MR-Egger and MR-PRESSO methods. More large populations are needed to confirm this MR association. Fourthly, the causal role of high circulating adiponectin in low BMD in our previous MR study^32^, but the detrimental change is not translated to the increase in osteoporotic fracture, and the related mechanisms are still not clear.

## Conclusion

In this two-sample MR study, high circulating adiponectin may be causally associated with reduced risk of type 2 diabetes and coronary artery disease, which may help prevent and treat these diseases.

## Supplementary Information

Supplementary Information 1.

Supplementary Information 2.

## Data Availability

Data supporting the findings of this study were available within the paper.
