# Supplementary material for: Causal associations of circulating adiponectin with cardiometabolic diseases and osteoporotic fracture
Source: Sci Rep. 2022 Apr 23;12:6689. doi: 10.1038/s41598-022-10586-1 (PMC9035157; doi:10.1038/s41598-022-10586-1)
Supplement: Supplementary file 1 — Supplementary Information 1. [file 41598_2022_10586_MOESM1_ESM.docx]

**
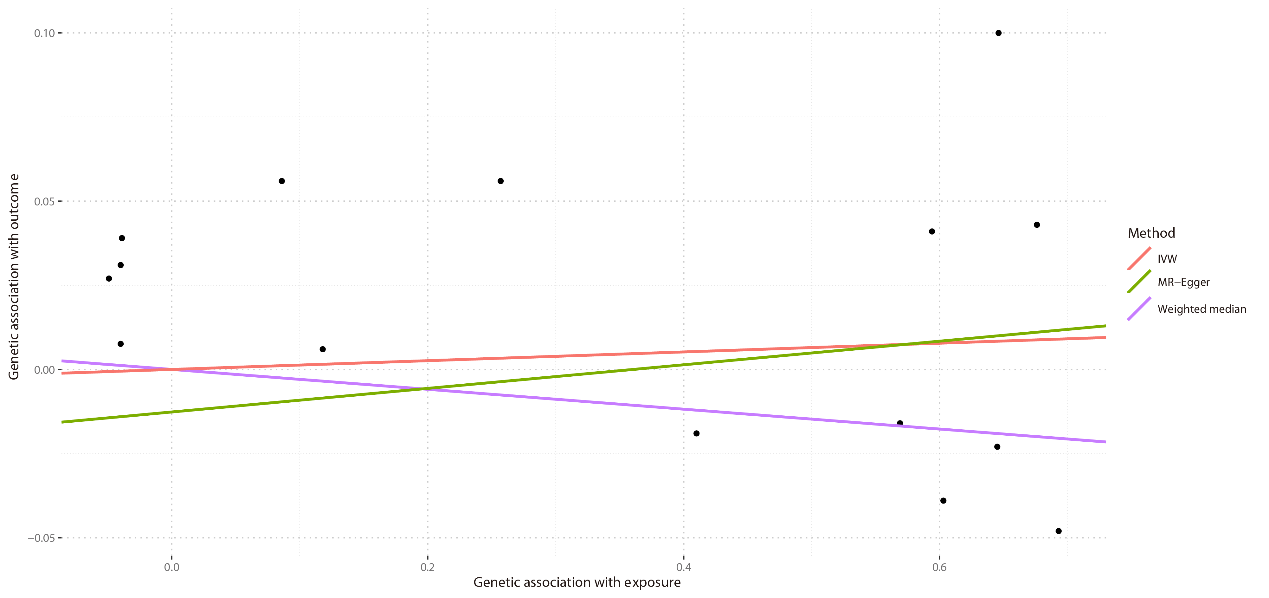
**

**Supplementary figure 1**

**
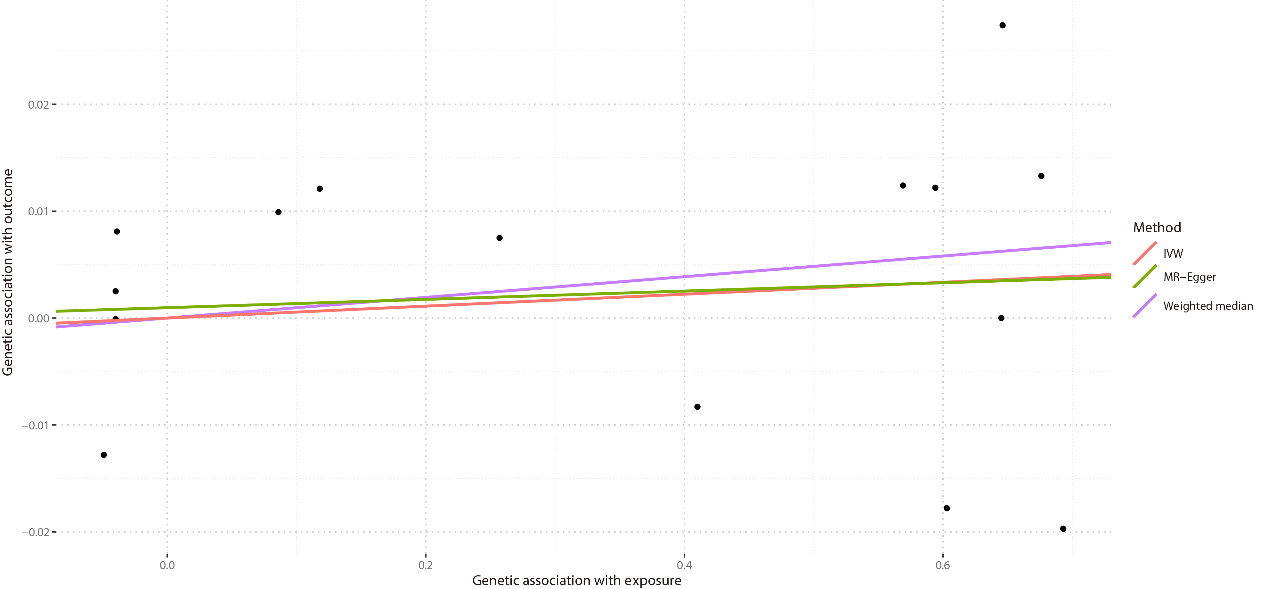
**

**Supplementary figure 2**

**
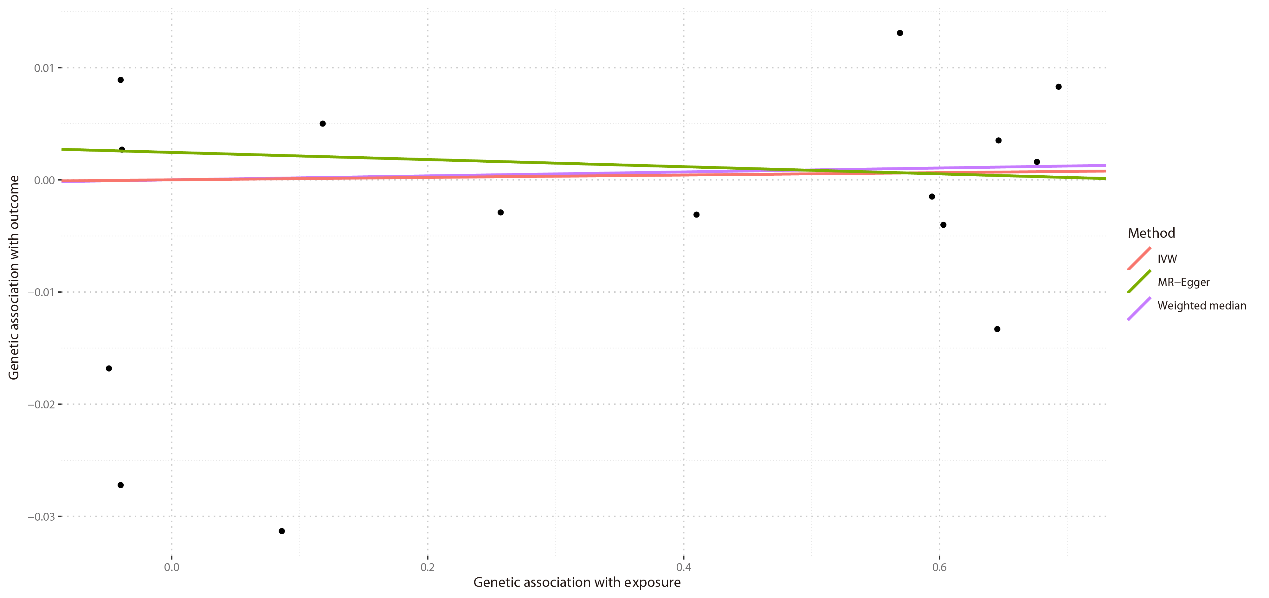
**

**Supplementary figure 3**

**
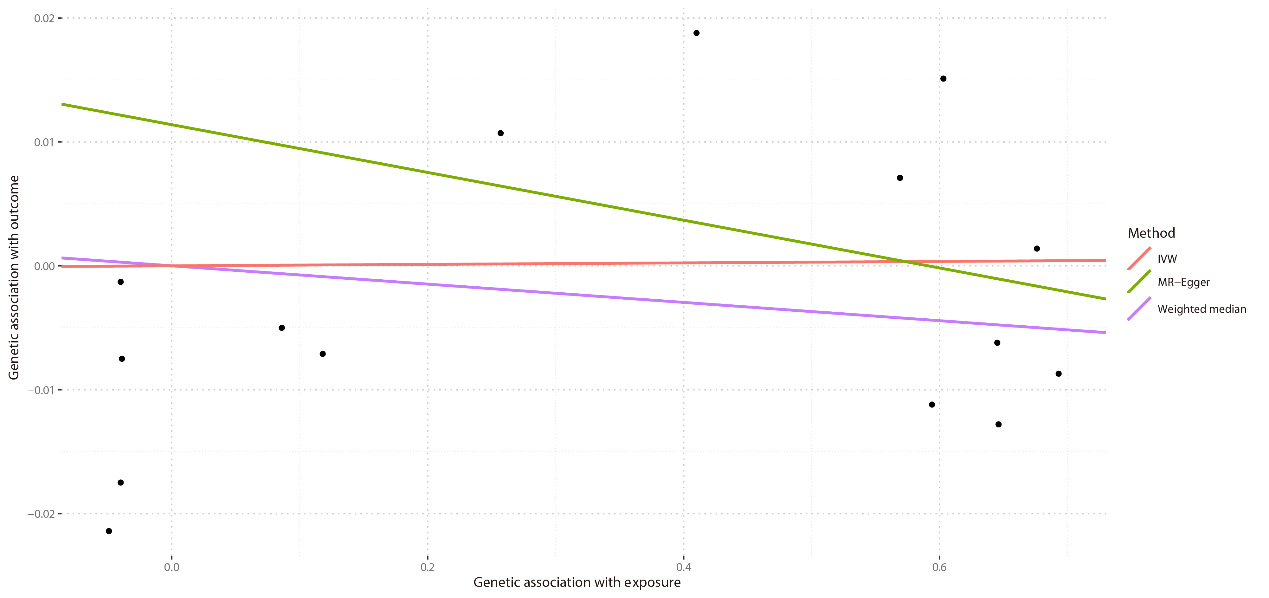
**

**Supplementary figure 4**

**
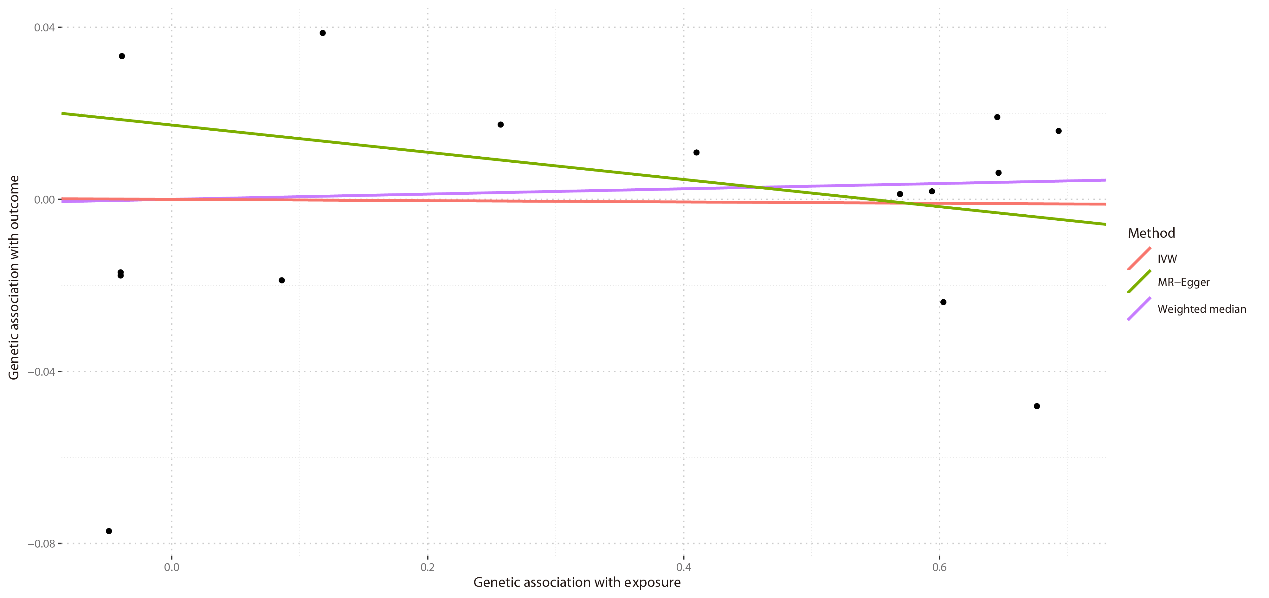
**

**Supplementary figure 5**

**
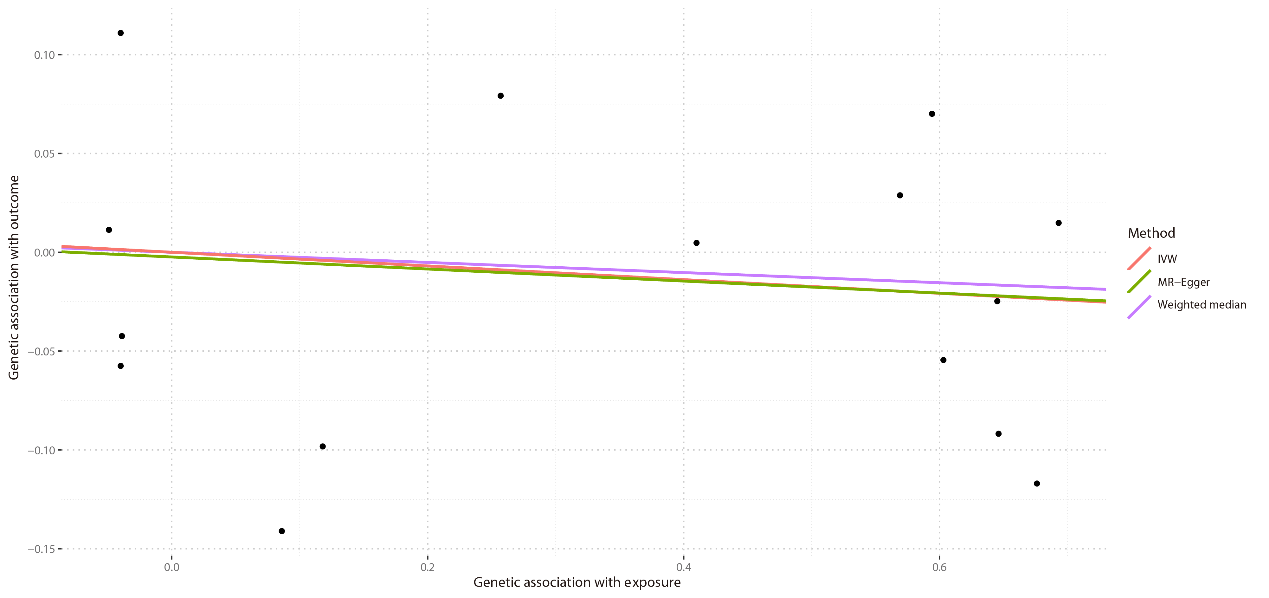
**

**Supplementary figure 6**

**
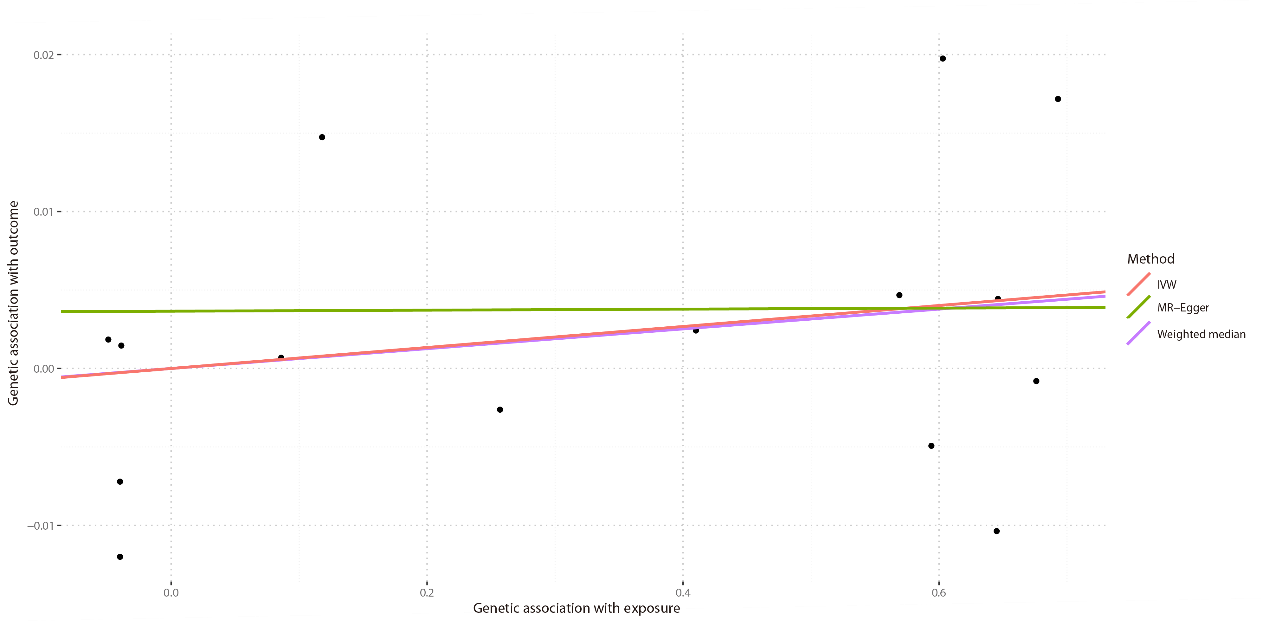
**

**Supplementary figure 7**

**Supplementary figure legend**

**Supplementary figure 1** Scatter Plots of the association between circulating adiponectin and Type 2 diabetes in MR analysis. Analyses were conducted using the IVW, MR-Egger and weighted median methods.

**Supplementary figure 2** Scatter Plots of the association between circulating adiponectin and coronary artery disease in MR analysis. Analyses were conducted using the IVW, MR-Egger and weighted median methods.

**Supplementary figure 3** Scatter Plots of the association between circulating adiponectin and heart failure in MR analysis. Analyses were conducted using the IVW, MR-Egger and weighted median methods.

**Supplementary figure 4** Scatter Plots of the association between circulating adiponectin and atrial fibrillation in MR analysis. Analyses were conducted using the IVW, MR-Egger and weighted median methods.

**Supplementary figure 5** Scatter Plots of the association between circulating adiponectin and cerebral ischemia in MR analysis. Analyses were conducted using the IVW, MR-Egger and weighted median methods.

**Supplementary figure 6** Scatter Plots of the association between circulating adiponectin and intracerebral hemorrhage in MR analysis. Analyses were conducted using the IVW, MR-Egger and weighted median methods.

**Supplementary figure 7** Scatter Plots of the association between circulating adiponectin and osteoporotic fracture in MR analysis. Analyses were conducted using the IVW, MR-Egger and weighted median methods.
